# Non-invasive and quantitive analysis of flatfoot based on ultrasound

**DOI:** 10.3389/fbioe.2022.961462

**Published:** 2022-09-06

**Authors:** Zhende Jiang, Qianpeng Zhang, Lei Ren, Zhihui Qian

**Affiliations:** ^1^ Key Laboratory of Bionic Engineering, Jilin University, Changchun, China; ^2^ Orthopaedic Medical Center, The Second Hospital of Jilin University, Changchun, China; ^3^ Department of Radiology, Second Hospital of Jilin University, Changchun, China; ^4^ School of Mechanical, Aerospace and Civil Engineering, University of Manchester, Manchester, United Kingdom

**Keywords:** flatfoot, plantar fascia angle, calcaneal pitch angle, medial cuneiform height, diagnosis

## Abstract

Flatfoot is a common foot deformity that seriously affects the quality of life. The aim of this study is to develop an accurate and noninvasive method for the diagnosis of flatfoot based on B-mode ultrasound. In this study, 51 patients (the flatfoot group) and 43 healthy subjects (the control group) were included. The plantar fascia angle, a new measurement for use in the diagnosis of flatfoot is proposed, as determined using B-mode ultrasound. For comparison, the calcaneal pitch angle and medial cuneiform height were also measured using lateral X-radiography, based on traditional diagnostic methods. The intraclass correlation values of the plantar fascia angle, the calcaneal pitch angle, and the medial cuneiform height were all more than 0.95, and there is a moderate correlation (r = 0.51) between the medial cuneiform height and the calcaneal pitch angle, and an excellent correlation (r = 0.85) between the plantar fascia angle and the calcaneal pitch angle. The optimal cutoff value, sensitivity, and specificity for medial cuneiform height in flatfoot diagnosis were 12.8 mm, 93.0%, and 54.9%, respectively. The optimal cutoff value, sensitivity, and specificity for plantar fascia angle in flatfoot diagnosis were 9.8°, 97.7%, and 94.1%, respectively. The proposed plantar fascia angle has good sensitivity and specificity in diagnosing flatfoot, therefore supplying a new approach for the noninvasive diagnosis of flatfoot.

## Introduction

The human foot is a complex system comprising 26 bones, 33 joints, and more than 100 muscles, tendons, and ligaments. It plays an important role in human weight-bearing and propulsion (Oleksy et al., 2010). Flatfoot is a common type of foot deformity, in which the foot has little or no arch (Sung, 2016). It has been confirmed that flatfoot is a complex deformity, that is, highly related to abnormal changes in the medial longitudinal arch (Pehlivan et al., 2009; Shibuya et al., 2010; Abousayed et al., 2017). Though a study showed that the quality of life seemed not to be influenced by the height of foot arch (Lopez-Lopez et al., 2018), many studies demonstrated that the lower foot arch of flatfoot can lead to abnormal gait and lower-limb alignment, affect the function of shock absorption, and lead to plantar fasciitis (PF), medial tibial stress syndrome, patellar tendon disease, and other problems, and seriously affect the quality of life (Kohls-Gatzoulis et al., 2004; Wearing et al., 2006; Levinger et al., 2010; Van der Worp et al., 2011; Hamstra-Wright et al., 2015).

Diagnostic methods of flatfoot mainly include physical examination and imaging examination (Abousayed et al., 2017). Physical examination is mainly carried out through visual examination, palpation, and mobility measurement. Visual examination mainly involves observing the shape and alignment of the foot; the most commonly used rating tools are the foot posture index (FPI-6) and footprints. FPI-6 allows the foot to be evaluated on three planes; it is composed of six separate evaluation parts, and summarizes the results to reflect the posture of the foot (Oleksy et al., 2010). Palpation is mainly carried out along the posterior tibial tendon to determine whether there is posterior tibial tendonitis or posterior tibial tendon rupture (Abousayed et al., 2017). Mobility measurement is mainly conducted to evaluate muscle strength and movement; for example, the dorsal flexion test is used to judge the tension of the gastrocnemius–soleus muscle complex and the heel lifting test is used to judge the function of the posterior tibial tendon (Abousayed et al., 2017). However, the results of physical examination will be affected by the subjective judgment of podiatrists, and it is troublesome to obtain and measure accurate footprints, so imaging diagnosis is necessary to further clarify the diagnosis and severity of the disease (Bock P, 2018).

The evaluation of X-radiographs of the weight-bearing foot and ankle is still the gold standard for the diagnosis of flatfoot and pes cavus (Abousayed et al., 2017). Anteroposterior X-radiography of the weight-bearing foot can show abduction of the forefoot and the uncovered talus. Many parameters are used to evaluate the uncovered talus, including the talus coverage angle, the percentage of uncovered talus, and the lateral discordant angle (Deland, 2008; Chan et al., 2015). The calcaneal pitch angle and medial cuneiform height of the talus can be measured using lateral X-radiographs of the weight-bearing foot (Younger et al., 2005; Bock et al., 2018). Magnetic resonance imaging and ultrasonography are mainly used for preoperative evaluation of the posterior tibial tendon, spring ligament, and plantar fascia, in order to optimize operation plans (Harish et al., 2008; Arnoldner et al., 2015; Abousayed et al., 2017). A great contribution of the ultrasound is that it opens the opportunity to measure parameters such as the size, shape, angle and biomechanical properties of the muscle, tendon, ligament and fascia (Romero-Morales et al., 2019; Schillizzi et al., 2020; Romero-Morales et al., 2021), of which the angle of the plantar fascia based on ultrasound was the specifically we are looking at in relation to flatfoot.

X-radiography is important in flatfoot diagnosis (Abousayed et al., 2017). However, X-radiation has potential radiological hazard (Prasarn, 2014). Therefore, the study of rapid, non-radiological, quantifiable and convenient methods for the diagnosis of flatfoot has important clinical significance and social value. As is known, the plantar fascia, as the main structure connecting the calcaneus and the proximal phalanges, maintains the shape and function of the longitudinal arch of the foot (Orchard, 2012; McKeon et al., 2015). When the medial longitudinal arch changes, the characteristics of the plantar fascia will change accordingly. At present, there are many reports on the characteristics of plantar fascia in flatfoot (Wang et al., 2019; Qian et al., 2021). However, to the authors’ knowledge, the characteristics of the plantar fascia angle (the angle between the plantar fascia and horizontal line) in flatfoot have not been reported to date.

Previous studies have shown that the calcaneal pitch angle and the medial cuneiform height are effective diagnostic methods for flatfoot (Bock et al., 2018; Flores et al., 2019). Therefore, this study takes the calcaneal pitch angle measured from X-radiographs of the lateral weight-bearing foot as the diagnostic gold standard; the plantar fascia angle in patients with flatfoot was measured to explore the relationship between the plantar fascia angle and the diagnosis of flatfoot. The value of the plantar fascia angle in diagnosing flatfoot was evaluated by comparing it with the medial cuneiform height. It is hypothesized that the plantar fascia angle would be influenced by the height of the arch, and the plantar fascia angle would be an effective method for the diagnosis of flatfoot. The purpose of this study is to propose a non-invasive method for flatfoot diagnosis based on ultrasound.

## Methods

### Ethics statement

This study was based on the principles outlined in the Declaration of Helsinki, and was approved by the Ethics Committee of the Second Hospital of Jilin University (No.2020085). All volunteers who participated in the study signed a written informed consent agreement.

### Patients

This is a prospective study. The sample size calculated by G-Power (Kang, 2021) was 22 when *α* = 0.05, power = 0.95, based on pre-experiments. The inclusion criteria of the experimental group were: ①patients treated in the outpatient center of the Second Hospital of Jilin University from April 2021 to December 2021; ② ages of the patients ≥ 18 years old; ③ the calcaneal pitch angle of the patients were less than 20°. Healthy adults with similar age, sex, height, and weight were selected as the control group. The exclusion criteria were: ①a history of foot trauma or surgery; ②a diagnosis of systemic disease, such as rheumatoid arthritis, diabetes, or gout; ③a diagnosis of local disease, such as plantar fibromatosis.

### Test device and procedure

**FIGURE 1:**
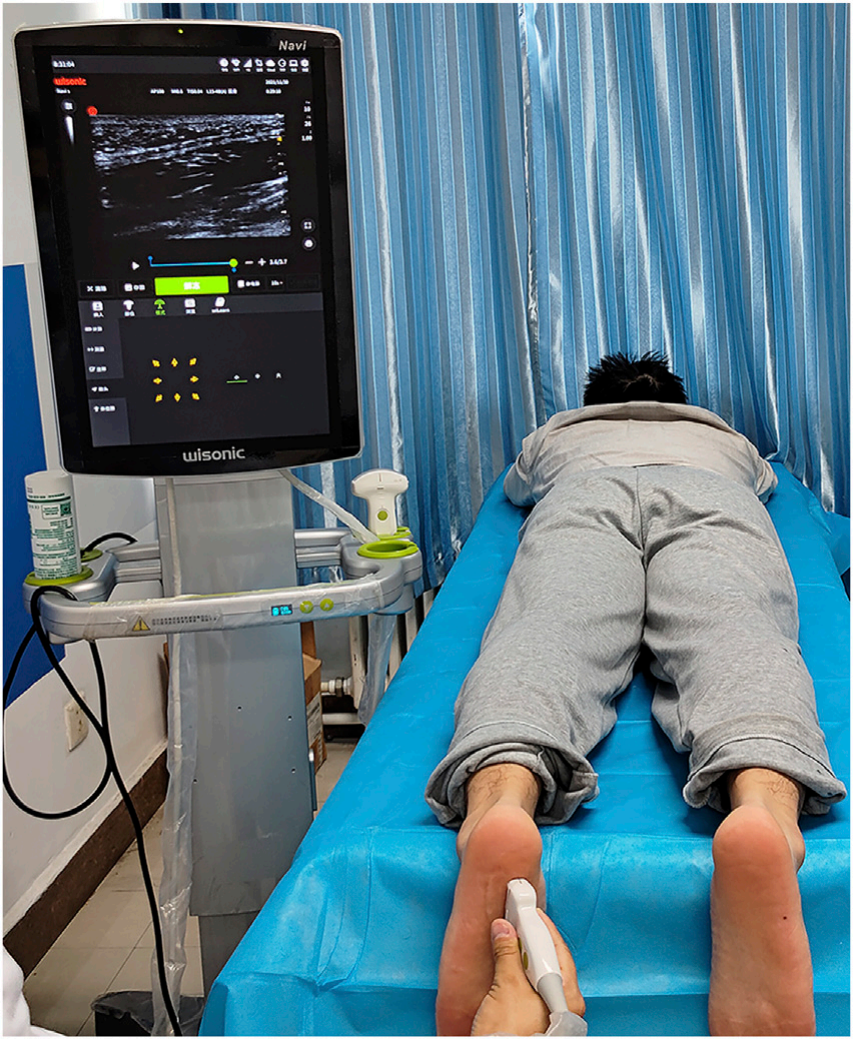
Position for ultrasound measurement of plantar fascia angle.

A Wisonic ultrasonic scanner (Navis, Wisonic) was used in B mode to measure the plantar fascia angle. The linear transducer frequency was 10–2 MHz. During measurement, each subject lay prone on the examination bed, with the lower limbs straight and the feet hanging over the edge of the examination bed in a neutral position (Haen et al., 2017) (Figure 1). The upper body and legs were relaxed.

The probe was placed under the navicular and medial cuneiform along the long axis of the plantar fascia, and the mark point of the probe was towards to the calcaneus, so that the proximal end of plantar fascia could be presented on the left side of the ultrasound image. In this study, the sampling depth was 3 cm, and the mechanical index was 0.7. The probe was gently placed on the plantar surface and two-dimensional ultrasound images were taken.

**FIGURE 2:**
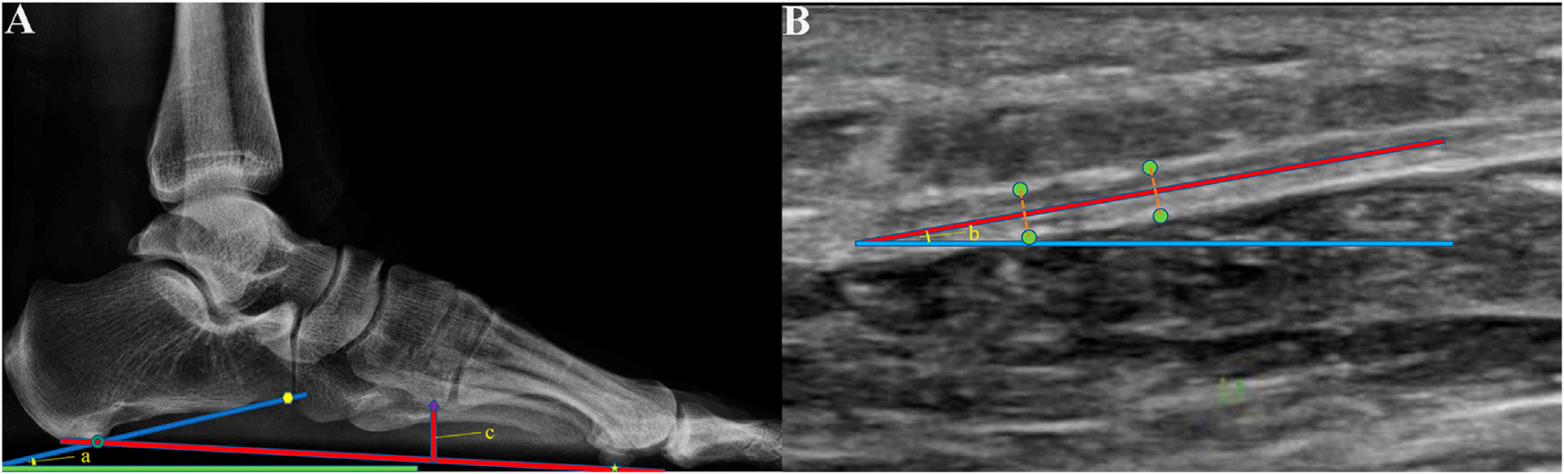
**(A)** Calcaneal pitch angle (angle a) and medial cuneiform height (c) were measured from X-radiographs of the weight-bearing foot. Calcaneal pitch angle is the angle between the supporting surface (green line) and the line (dark blue line) connected the lowest point of calcaneus (dark green circle) and the lowest point of the anterior edge of the calcaneus (yellow point). Medial cuneiform height is the distance from the lowest point of the medical cuneiform (the purple rhombic point) to the line that connected the lowest point of calcaneus (dark green circle) and the lowest point of the sesamoid under the first metatarsal (yellow five-point star). **(B)** Plantar fascia angle (angle b) was measured using B-mode ultrasound. It was defined as the angle between the middle line (red line) of the plantar fascia and the horizontal line (the blue line parallel to the probe and skin). The green points were the edge of the plantar fascia.

After ultrasonic examination, X-radiography of the weight-bearing foot was performed by an experienced radiologist. The calcaneal pitch angle and medial cuneiform height were measured from the lateral X-radiograph (Figure 2A) by an experienced radiologist. The calcaneal pitch angle is the angle between a line drawn along the most inferior part of the calcaneus and the supporting surface. (Abousayed et al., 2017) The medial cuneiform height is the distance from the lowest point of medial cuneiform to the line that connect the lowest point of calcaneus and the lowest point of the sesamoids of the first metatarsal (Bock P, 2018). The plantar fascia angle (Figure 2B) is defined as the angle between the plantar fascia and the horizontal line (the line parallel to the probe and skin), which was measured by an experienced ultrasonographer, who was unknown to the results of X-Ray. The plantar fascia angle, calcaneal pitch angle, and medial cuneiform height were measured three times each, to ensure reproducibility.

### Statistical analysis

The data were analyzed using IBM Statistical Package for the Social Sciences (SPSS) version 26.0 (SPSS Inc., IL). Continuous variables (age, height, weight, calcaneal pitch angle, plantar fascia angle, and medial cuneiform height) were expressed as mean ± standard deviation. The χ^2^ test was used to analyze differences between the sexes in the flatfoot and control groups. Firstly, intraclass correlation (ICC) analysis of plantar fascia angle, calcaneal pitch angle, and medial cuneiform height was conducted, and the 95% confidence intervals (95% CIs) and ICC values were calculated. Secondly, Pearson correlation analysis was conducted between the calcaneal pitch angle, plantar fascia angle, and medial cuneiform height. The absolute value of the Pearson correlation coefficient (*r*) was classified as poor (0.00 ± 0.20), fair (0.21 ± 0.40), moderate (0.41 ± 0.60), good (0.61 ± 0.80), or excellent (0.81 ± 1.00) (Landis JR, 1977). Thirdly, receiver operating characteristic (ROC) curves for all parameters were obtained to calculate sensitivity, specificity, area under the curve (AUC), and optimal cutoff value. The AUCs were compared using the *Z* test. Finally, sex, age, side (left or right) and PF (With or Without) were set as classification parameters to compare their influences on plantar fascia angle and medial cuneiform height. Statistical significance was considered for *p* < 0.05.

## Results

### Basic characteristics

51 patients with flatfoot and 43 healthy subjects were included in the study. Firstly, the basic characteristics of the two groups were compared and analyzed; the results are shown in Table 1. The χ^2^ test was used for sex comparison, and the independent sample *t* test was used for comparisons of age, height, and weight. Values of *p* are all greater than 0.05. That is, there was no statistical difference in sex, age, height, and weight between the flatfoot group and the control group.

**TABLE 1:** Basic characteristics of flatfoot and control groups.

Basic characteristic	Flatfoot group	Control group	*P*
Sex (M/F)	23/28	24/19	0.301
Age (years)	39.81 ± 13.983	35.81 ± 13.162	0.161
Height (cm)	168.27 ± 7.228	169.21 ± 6.236	0.135
Weight (kg)	65.45 ± 10.473	65.02 ± 8.651	0.080

### Intraclass correlation analysis

Intraclass correlation analysis of plantar fascia angle, calcaneal pitch angle, and medial cuneiform height was conducted; the results are shown in Table 2. The ICC values of the three parameters are all greater than 0.9; this indicates that the three parameters had good intra-observer reproducibility.

**TABLE 2:** Intraclass correlation (ICC) values and 95% confidence intervals (95% CIs) of the measured parameters.

Parameter	ICC	95% CI
Plantar fascia angle	0.973	(0.962, 0.981)
Calcaneal pitch angle	0.993	(0.989, 0.995)
Medial cuneiform height	0.982	(0.975, 0.988)

### Pearson correlation analysis

**FIGURE 3:**
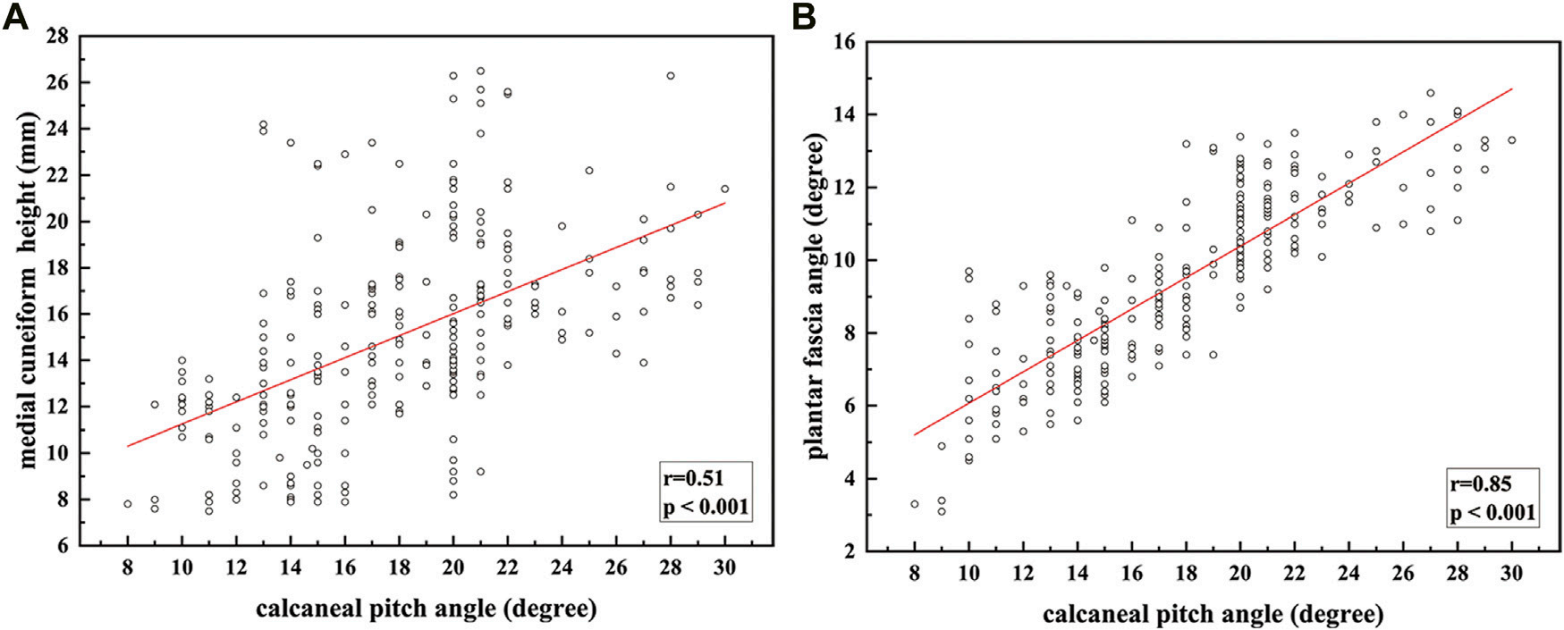
Fitting relationship between calcaneal pitch angle and: **(A)** medial cuneiform height; **(B)** plantar fascia angle.

Figure 3A is a scatter plot of medial cuneiform height with respect to calcaneal pitch angle for all subjects; Figure 3B is a scatter plot of plantar fascia angle with respect to calcaneal pitch angle for all subjects. There is a moderate correlation (*r* = 0.51, *p* < 0.001) between medial cuneiform height and calcaneal pitch angle; the correlation between plantar fascia angle and calcaneal pitch angle is excellent (*r* = 0.85, *p* < 0.001).

### Diagnostic performance evaluation

**FIGURE 4:**
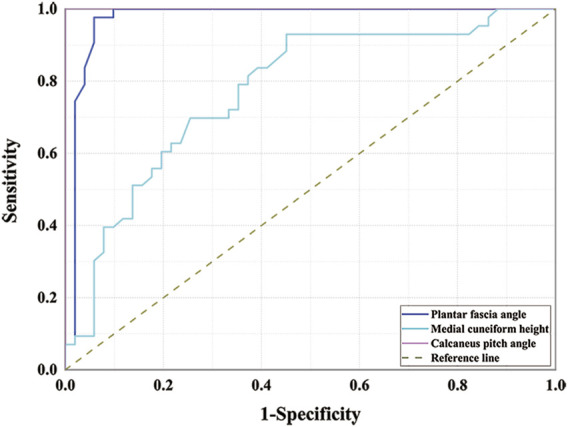
Receiver operating characteristic curves for medial cuneiform height and plantar fascia angle.

The ROC curves for the medial cuneiform height and the plantar fascia angle are shown in Figure 4. The AUC for the medial cuneiform height is 0.775 (0.679–0.871); that for the plantar fascia angle is 0.973 (0.935–1.000). The optimal cutoff value, sensitivity, and specificity for the medial cuneiform height in flatfoot diagnosis are 12.8 mm, 93.0%, and 54.9%, respectively; the optimal cutoff value, sensitivity, and specificity for the plantar fascia angle in flatfoot diagnosis are 9.8°, 97.7%, and 94.1%, respectively. The results for the AUC were compared using the *Z* test. The AUC for the plantar fascia angle is more significant than that for the medial cuneiform height (*Z* = 2.55, *p* = 0.0108); this suggests that the plantar fascia angle has better diagnostic efficiency in the diagnosis of flatfoot. The AUC for the plantar fascia angle is not more statistically significant than that for the calcaneal pitch angle (*Z* = 1.42, *p* = 0.1556); this suggests that the plantar fascia angle has the same diagnostic efficiency as calcaneal pitch angle in the diagnosis of flatfoot.

### Influences factors

The influences of sex, age, and side (left or right) on plantar fascia angle and medial cuneiform height are shown in Table 3. There was no difference in sex and side (*p* > 0.05), while statistical differences were found between groups of different ages in plantar fascia angle. Between subjects younger and older than 40 years, the value of *p* was 0.023. Between subjects younger and older than 50 years, the value of *p* was 0.001. These results show that the plantar fascia angle is decreased for subjects older than 40 years.

**TABLE 3:** The influences of sex, age, side (left or right) on plantar fascia angle and medial cuneiform height.

Group		Plantar fascia angle (degree)	*P*	Medial cuneiform height (mm)	*P*
Sex	Female (47)	9.20 ± 2.30	0.197	14.33 ± 3.95	0.090
	Male (47)	9.81 ± 2.31		15.82 ± 4.47	
Side	Left (41)	9.78 ± 2.41	0.318	15.08 ± 3.71	0.982
	Right (53)	9.29 ± 2.24		15.06 ± 4.68	
Age	<30 y (31)	9.55 ± 2.13	0.889	15.04 ± 5.53	0.970
	≥30 y (63)	9.48 ± 2.42		15.08 ± 3.53	
	<40 y (52)	9.99 ± 2.09	0.023*	15.51 ± 4.61	0.269
	≥40 y (42)	8.90 ± 2.46		14.53 ± 3.77	
	<50 y (68)	10.00 ± 2.20	0.001*	15.35 ± 4.26	0.312
	≥50 y (26)	8.21 ± 2.14		14.35 ± 4.27	

*Difference was statistically significant.

The influence of PF was shown in Table 4. The results showed that the plantar fascia angle would not be influenced by PF both in the flatfoot group and the healthy control group.

**TABLE 4:** The influences of plantar fasciitis on plantar fascia angle.

Group		Plantar fascia angle (degree)	*P*
Flatfoot (51)	With PF (9)	8.09 ± 1.28	0.386
	Without PF (42)	7.77 ± 1.82	
Healthy Control (43)	With PF (5)	11.53 ± 0.75	0.903
	Without PF (38)	11.49 ± 1.26	

PF is the abbreviation of plantar fasciitis.

## Discussion

Flatfoot is a common foot disease, which can seriously affect the quality of life (Kohls-Gatzoulis et al., 2004; Wearing et al., 2006; Levinger et al., 2010; Van der Worp et al., 2011; Hamstra-Wright et al., 2015). At present, X-radiography of the weight-bearing foot is still the gold standard for the diagnosis of flatfoot (Abousayed et al., 2017). However, radiological diagnosis has led to a 600% increase in medical radiation exposure of the United States population (Linet et al., 2012). X-radiation is a known carcinogen that can cause malignancy (Prasarn, 2014), which can be accumulated (Giordano et al., 2009; Giordano et al., 2011; Taher et al., 2013). Thus, exploration of a nonionizing examination method is of great significance in the clinical field. In this study, ultrasound was used to measure the angle of the plantar fascia for the diagnosis of flatfoot. The calcaneal pitch angle measured from a lateral X-radiograph of the weight-bearing foot was used as a gold standard, and the diagnostic effect of the plantar fascia angle was studied and compared with the diagnostic efficiency of medial cuneiform height.

According to the results, there is no statistical difference in the basic characteristics between the flatfoot group and the control group (*p* > 0.05). Analysis of the measured results showed that the ICC values are all greater than 0.95, indicating that the values of the three parameters have good intra-observer reproducibility. Studies have also shown that the calcaneal pitch angle and the medial cuneiform height have a high degree of reliability between observers (Bock et al., 2018). Pearson correlation analysis was conducted between the medial cuneiform height, plantar fascia angle, and calcaneal pitch angle; the results show an excellent correlation between plantar fascia angle and calcaneal pitch angle (*r* = 0.85), better than that between medial cuneiform height and calcaneal pitch angle (*r* = 0.51).

In addition, the area under the ROC curve was used to test the diagnostic efficiency. The AUCs for the medial cuneiform height and plantar fascia angle are 0.775 and 0.973, respectively. Both of these measures have good diagnostic effect. The AUC for the plantar fascia angle is more significant than that for the medial cuneiform height (*Z* = 2.55, *p* = 0.0108); this suggests that the plantar fascia angle has better diagnostic efficiency in the diagnosis of flatfoot. The AUC for the plantar fascia angle is not more statistically significant than that for the calcaneal pitch angle (*Z* = 1.42, *p* = 0.1556); this suggests that the plantar fascia angle has the same diagnostic efficiency as calcaneal pitch angle in the diagnosis of flatfoot.

The sensitivity and specificity of the two methods for the diagnosis of flatfoot were calculated using the Youden index. The results show that the optimal cutoff value of plantar fascia angle to diagnose flatfoot is 9.8°; that is, when the measured plantar fascia angle is less than 9.8°, a diagnosis of flatfoot is indicated. The sensitivity and specificity of using this value to diagnose flatfoot are 97.7 and 94.1%, respectively. The optimal cutoff value of medial cuneiform height to diagnose flatfoot is 12.8 mm; that is, when the measured medial cuneiform height is less than 12.8 mm, flatfoot can be diagnosed. The sensitivity and specificity of using this value to diagnose flatfoot are 93.0 and 54.9%, respectively. The specificity of the plantar fascia angle is greater than that of medial cuneiform height. The specificity of the medial cuneiform height might be low because the medial cuneiform height is likely to be influenced by the varus and valgus of the foot.

The subject’s sex and the side of the affected foot did not influence the results for plantar fascia and medial cuneiform height (*p* > 0.05). However, the plantar fascia angle was affected in subjects older than 40 years. The results show statistical differences in plantar fascia angle between groups of different ages. Between subjects younger and older than 40 years, the value of *p* was 0.023. Between subjects younger and older than 50 years, the value of *p* was 0.001. Tas and Cetin (Tas and Cetin, 2019) also report that age is a potential parameter that might affect the morphologies and mechanical properties of plantar muscles. Changes in morphology and mechanical properties of plantar muscles would influence the medial longitudinal arch, leading to a change in the plantar fascia angle. In addition, the results of the study showed that the plantar fascia angle was not affected by plantar fasciitis. It may be for the reason that the plantar fasciitis was always happened at the insertion portion of plantar fascia (Orchard, 2012), while the plantar fascia angle was measured at the portion of the plantar fascia under the navicular and medial cuneiform, so plantar fascia angle would not be influenced by plantar fasciitis.

Ultrasound is a good tool in disease diagnosis; it is convenient, low-cost, and nonionizing. In the diagnosis of flatfoot, the plantar fascia angle measured using B-mode ultrasound has good sensitivity and specificity, as well as good intra-observer reproducibility. At the same time, it is portable and noninvasive; thus, it is more applicable in the diagnosis of flatfoot for disabled patients and children.

There are limitations in this study. Firstly, the flatfoot in this study was not graded according to severity. The role of plantar fascia angle in diagnosing the severity of flatfoot needs further study. Secondly, flatfoot can present different clinical manifestations, such as with or without hindfoot valgus, forefoot abduction, etc. (Hillstrom et al., 2013). These different clinical manifestations may influence the test results; this is to be considered in future work. Thirdly, the different conditions of the ankle/foot in X-ray (weight-bearing) and ultrasound (non-weight-bearing) would be a limitation for this study. However, even under the condition of non-weight-bearing, the plantar fascia angle still showed significant statistical difference between flatfoot (7.83 + 1.74°) and the healthy control (11.49 + 1.21°), *p* < 0.001; In addition, the plantar fascia angle showed an excellent correlation with calcaneal pitch angle and the sensitivity and specificity in diagnosing flatfoot is also excellent.

## Conclusion

Plantar fascia angle has an excellent sensitivity and specificity in diagnosing flatfoot, and there is good intra-observer reproducibility within this study, thus, it could seem to be an effective method to diagnose flatfoot, especially when it was used for flatfoot screening. However, further studies in larger populations with different flatfoot grades and different clinical manifestations are warranted to confirm these promising results.

## Data Availability

The original contributions presented in the study are included in the article/supplementary material, further inquiries can be directed to the corresponding authors.

## Abbreviations

PF, plantar fasciitis; FPI-6, foot posture index; ICC, intraclass correlation; 95% CIs, 95% confidence intervals; ROC, receiver operating characteristic; AUC, area under the curve.
